# A technical note on contamination from PRF tubes containing silica and silicone

**DOI:** 10.1186/s12903-021-01497-0

**Published:** 2021-03-19

**Authors:** Richard J. Miron, Tomoyuki Kawase, Anika Dham, Yufeng Zhang, Masako Fujioka-Kobayashi, Anton Sculean

**Affiliations:** 1 Department of Periodontology, University of Bern, Bern, Switzerland; 2 Division of Oral Bioengineering, Institute of Medicine and Dentistry, Niigata University, Niigata, Japan; 3 Department of Cranio-Maxillofacial Surgery, Inselspital, Bern University Hospital, University of Bern, Bern, Switzerland; 4 Department of Oral Implantology, University of Wuhan, Wuhan, China

**Keywords:** Platelet rich fibrin, Platelets, A-PRF, I-PRF, L-PRF

## Abstract

**Background:**

Platelet-rich fibrin (PRF) has been widely utilized in modern medicine and dentistry owing to its ability to rapidly stimulate neoangiogenesis, leading to faster tissue regeneration. While improvements over traditional platelet rich plasma therapies (which use chemical additives such as bovine thrombin and calcium chloride) have been observed, most clinicians are unaware that many tubes utilized for the production of ‘natural’ and ‘100% autologous’ PRF may in fact contain chemical additives without appropriate or transparent knowledge provided to the treating clinician. The aim of this overview article is therefore to provide a technical note on recent discoveries related to PRF tubes and describe recent trends related to research on the topic from the authors laboratories.

**Methods:**

Recommendations are provided to clinicians with the aim of further optimizing PRF clots/membranes by appropriate understanding of PRF tubes. The most common additives to PRF tubes reported in the literature are silica and/or silicone. A variety of studies have been performed on their topic described in this narrative review article.

**Results:**

Typically, PRF production is best achieved with plain, chemical-free glass tubes. Unfortunately, a variety of other centrifugation tubes commonly used for lab testing/diagnostics and not necessarily manufactured for human use have been utilized in clinical practice for the production of PRF with unpredictable clinical outcomes. Many clinicians have noted an increased variability in PRF clot sizes, a decreased rate of clot formation (PRF remains liquid even after an adequate protocol is followed), or even an increased rate in the clinical signs of inflammation following the use of PRF.

**Conclusion:**

This technical note addresses these issues in detail and provides scientific background of recent research articles on the topic. Furthermore, the need to adequately select appropriate centrifugation tubes for the production of PRF is highlighted with quantitative data provided from in vitro and animal investigations emphasizing the negative impact of the addition of silica/silicone on clot formation, cell behavior and in vivo inflammation.

## Background

Platelet-rich fibrin (PRF) has been widely utilized as an autologous regenerative modality for over 2 decades demonstrating positive outcomes on tissue regeneration. Platelet rich plasma (PRP) was first introduced over 20 years ago with the aim of concentrating platelets from whole blood [1–5]. It was later revealed that PRP contains an array of naturally derived growth factors including platelet-derived growth factor (PDGF), vascular endothelial growth factor (VEGF), transforming growth factor beta (TGF-β), amongst others. Over the years PRP has been shown to facilitate new blood vessel formation (angiogenesis) and responsible for the rapid recruitment of cells and their proliferation and/or differentiation into various cell types [1–5]. While PRP continues to be heavily utilized in many fields of regenerative medicine, one of its major and reported disadvantages has been its inclusion of anticoagulants; known suppressors of clotting (clotting is an important first step during the wound healing cascade) [1, 6, 7]. Nevertheless, the simplicity and low cost of harvesting peripheral blood and their concentrations into platelet concentrates led to the fast and easy-to-obtain growth factor delivery system utilized commonly in many fields of tissue regeneration [8, 9].

PRP excelled in 2 main areas: 1) it could be utilized with extended centrifugation protocols owing to the lack of clot formation resulting from the use of anticoagulants, and 2) most of the kits were specific to certain centrifugation devices; therefore, all kits and centrifugation tubes were packaged individually for single use and most often with appropriate FDA-/CE-clearance. Since then, however, PRF was introduced as a second-generation platelet concentrate with the main highlighted advantage of being ‘100% autologous’ and utilizing completely ‘plain/additive-free’ tubes. In initial publications on PRF, clotting occurred when plain glass tubes were used to activate the intrinsic coagulation cascade [10]. Today, however, a variety of commercially available glass tubes (with or without chemical additives) and silica-coated plastic tubes have been utilized with varying degrees of success. Owing to the increased use of PRF in clinical practice, a variety of additional ‘commercially available’ tubes have been brought to market and utilized for the production of PRF, many without appropriate regulatory approval. Such tubes are generally only approved by regulatory authorities for laboratory diagnostic use [10]. Their safety in PRF therapy and human use has recently been put into question, as little background knowledge on the topic has been provided to clinicians [10].

The aim of this overview narrative article was therefore to provide a technical note on recent discoveries related to PRF tubes and describe recent trends related to research on the topic from the authors laboratories. Recommendations are provided to clinicians with the aim of further optimizing PRF clots/membranes by appropriate selection and understanding of PRF tubes.

## Main text

### Study 1: Miron et al. (2019)—the size of PRF membranes is dependent on centrifugation tubes and not centrifugation devices

The aim of this study was to compare and contrast the effect of 3 different centrifugation devices (IntraSpin, Process for PRF and Salvin) on the final outcomes of PRF clots [11]. In this study, 2 centrifugation speeds were utilized for each device to compare and contrast the differences between centrifugation devices. One of the most surprising observations, however, was the fact that PRF membranes produced using glass tubes (BD-Salvin and Process for PRF) were larger than those produced using IntraSpin plastic tubes. Clot size varied by as much as 200%, whereas the differences between the actual centrifugation devices was minimal. This clearly indicated that PRF tubes seemed to have a greater effect on the final size outcomes of PRF clots than PRF centrifugation devices [11].

To directly investigate the effects of PRF tubes on the final outcomes of PRF clot sizes, 2 tubes from each of the manufacturers (IntraSpin, Salvin and Process for PRF) were collected from a single participant in a random order (6 tubes total) [11]. Thereafter, each of the 6 tubes was placed into one centrifugation device. By utilizing such an approach, we could precisely address the role of PRF tubes by specifically introducing only one variable by utilizing exactly the same centrifugation device at the same speed with the same patient blood. It therefore became possible to truly assess the effect of PRF tube on the final PRF outcome. For each single participant, this mini study was repeated a total of 3 times (on each of the 3 centrifugation devices) for a total of 18 tubes harvested from each patient. Each series of 6 tubes was placed into each centrifugation device, including the IntraSpin Process for PRF and Salvin devices.

**Fig. 1:**
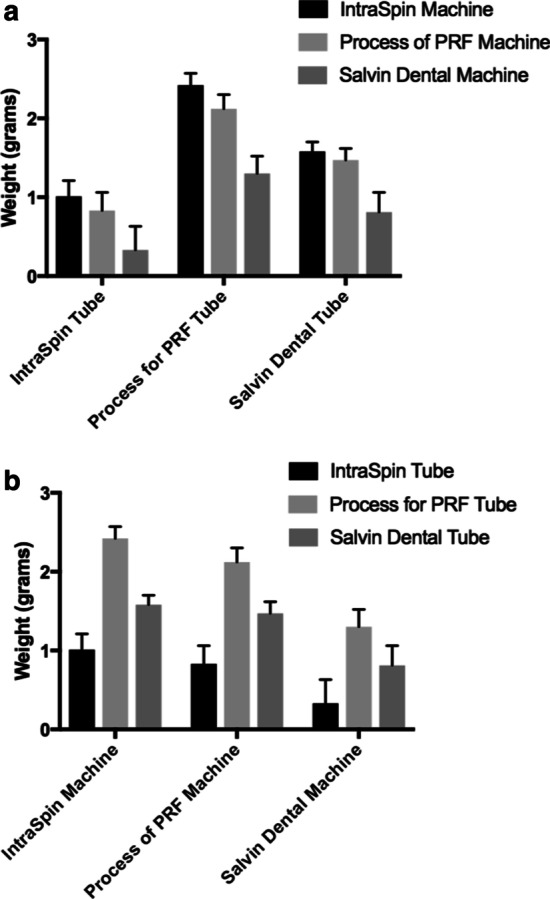
Final sizes of PRF clots produced utilizing 3 different centrifugation tubes in each of 3 different centrifugation devices (**a**) total of 9 tested groups). (A) Final PRF clot weight sizes depicted when different groups of tubes are used. (B) Final PRF clot weight sizes depicture when different centrifugation systems were utilized. Notice that in general, the IntraSpin centrifugation device produced slightly large clots (roughly 15%), whereas the glass tubes (Process for PRF, Salvin) produced the largest PRF clots (roughly 200–250% larger than those produced with plastic silica-coated IntraSpin tubes). Reprinted with permission from Miron et al. 2019 [11]

Interestingly, the PRF centrifugation device had little effect on the final size outcomes of PRF membranes (~ 15% differences between various fixed-angle centrifuges) (Fig. 1a, b). However, the differences in the PRF clots produced in the different tubes had a marked and pronounced effect on the final size outcomes of PRF tubes. Most surprisingly, it was observed that the IntraSpin tubes produced an approximately 200–250% smaller PRF clot than the other glass tubes. Furthermore, (and until then overlooked), it was revealed for the first time that the centrifugation tubes are central to the quality production of PRF. Future research investigating tube characteristics thus became critically important for the future optimization of PRF [11].

Clinical significance: In general, PRF clots produced in glass tubes produced larger membranes than silica-coated plastic tubes, and the selection of proper tubes had a much greater impact than the centrifugation device.

### Study 2: Tsujino et al. (2019)—evidence for contamination of silica microparticles in advanced platelet-rich fibrin matrix prepared using silica-coated plastic tubes

**Fig. 2:**
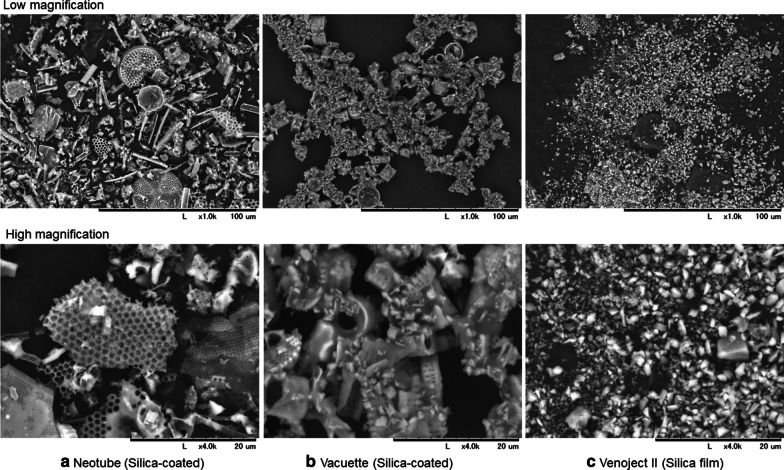
In this experiment, PRF clots were produced in 3 different commercially available tubes containing silica. Following centrifugation, clots were removed, the PRF clots were enzymatically digested, and ‘leftover’ remaining silica particles were visually assessed by scanning electron microscopy (SEM). SEM observations of silica microparticles contained in **a** Neotubes, **b** Vacuette tubes and **c** Venoject II tubes at low (top) and high magnification (bottom). Note the high incorporation of silica microparticles detached from PRF tube walls into PRF clots. Reprinted with permission from Tsujino et al. 2019 [10]

Simultaneously with the previous study, a group of authors based in Japan aimed to better understand the effects of silica-coated plastic tubes and, more specifically, the effects of silica release and incorporation into the PRF matrix [10]. In this study, blood samples were collected into 3 different brands of silica-containing plastic tubes and were immediately centrifuged as per the protocol for advanced PRF (A-PRF). A-PRF-like matrices were fixed and examined using scanning electron microscopy (SEM) or enzymatically degraded to spectrophotometrically determine the amount of silica microparticles. Regardless of tube brands and individual donors, both SEM examination and spectrophotometric determination demonstrated that significant levels of silica microparticles (between 5 and 30%) were incorporated into the A-PRF-like matrix, which would be consequently incorporated into human tissues within implantation sites (Fig. 2). Following enzymatic digestion of PRF clots, it was extremely apparent/obvious that the silica from the tube walls of plastic tubes, such as IntraSpin, was shed from the walls and incorporated within the PRF clots, which left quite significant byproducts within PRF clots, as observed via SEM in Fig. 2. It was reported by Tsujino et al. that clinicians should not exclude the possibility that silica microparticles negatively influence tissue regeneration, and the authors further recommend not using silica-containing tubes until their safety is assured.

Clinical significance: In general, tubes that contain silica-coating actually shed their coatings into PRF membranes, thus embedding silica particles at quite significant levels within PRF clots.

### Study 3: Masuki et al. (2019) Acute cytotoxic effects of silica microparticles used for coating plastic blood-collection tubes on human periosteal cells

In a second study by the same group, the effect of silica microparticles released from PRF tube walls was then investigated on human periosteal cells for potential cytotoxicity [12]. To further assess the biosafety of the silica microparticles, the authors examined their effect on primary human periosteal cells derived from alveolar bone. Silica microparticles were obtained from silica-coated tubes and added to cell cultures. Cellular responses were monitored using a tetrazolium assay, phase-contract inverted microscopy, an immunofluorescence method, and scanning electron microscopy [12].

**Fig. 3:**
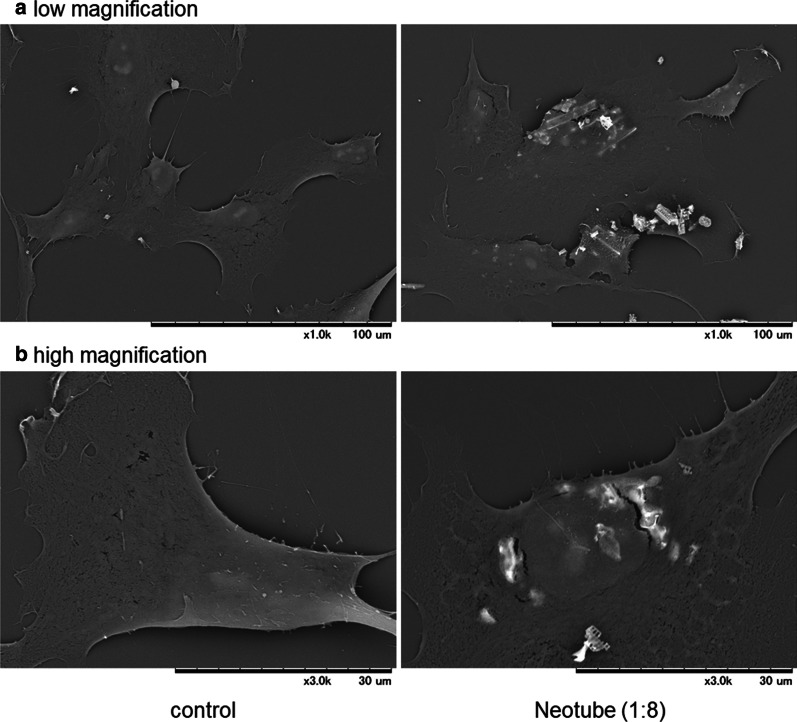
Microstructural images of human periosteal cells treated with silica microparticles. The cells treated with silica microparticles derived from Neotubes (1:8 dilution) for 24 h were fixed and examined using SEM at **a** low magnification and **b** high magnification. Note that the cells rapidly incorporated silica with high affinity. Similar observations were obtained from four other independent experiments, including those involving Vacuette’s silica. Reprinted with permission from Masuki et al. 2020 [12]

**Fig. 4:**
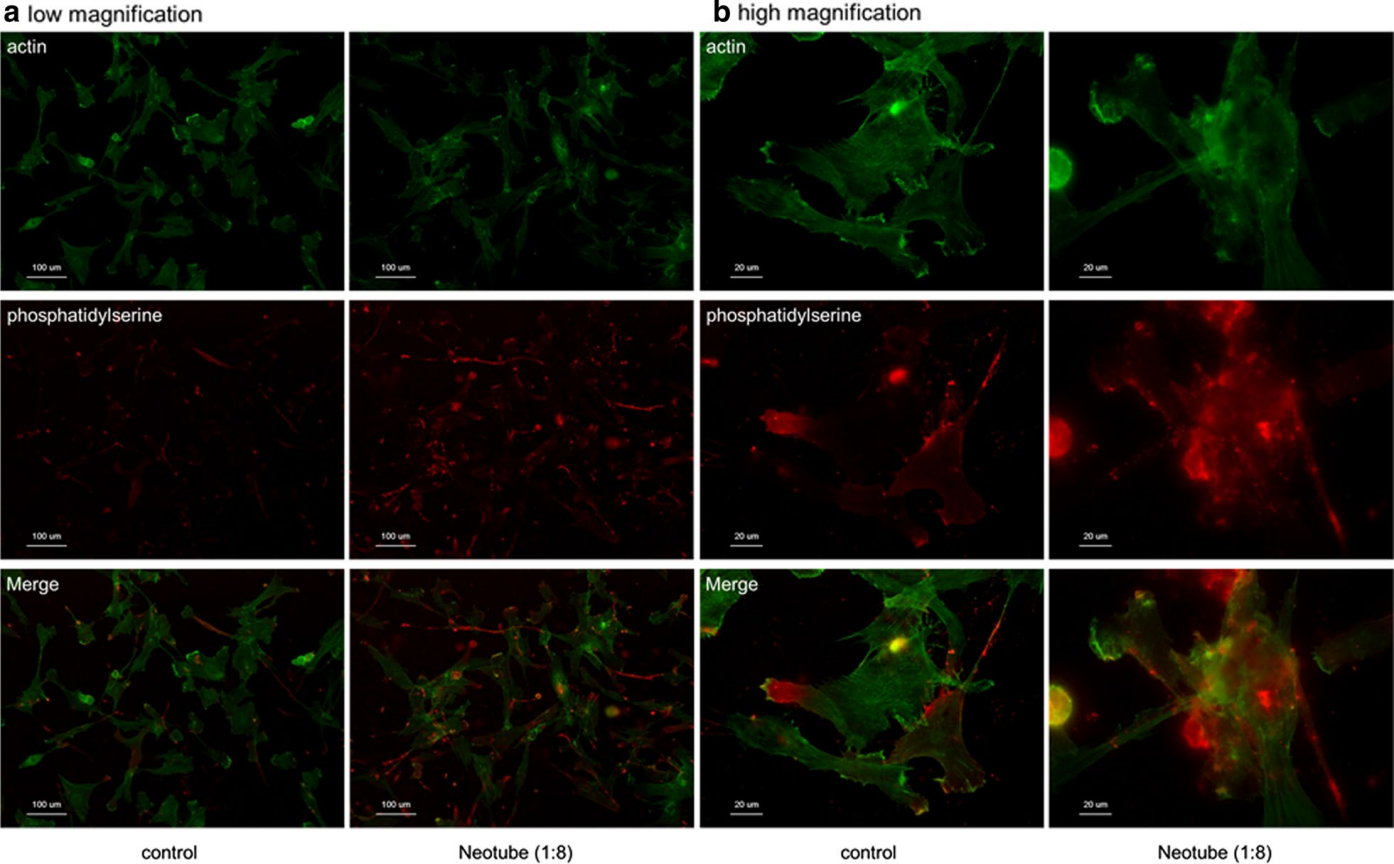
Fluorescence visualization of apoptosis in human periosteal cells treated with silica microparticles. The cells were treated with silica microparticles derived from Neotubes for 24 h. The fixed cells were probed with PE-conjugated annexin V for detection of phosphatidylserine on the cell surface, which is accepted as a marker of apoptosis, and are shown at **a** low magnification and **b** high magnification. The cells were counterstained with FITC-conjugated phalloidin to visualize cytoskeletal polymerized actin. Similar observations were obtained from four other independent experiments, including those involving Vacuette’s silica. Reprinted with permission from Masuki et al. 2020 [12]

It was found that the silica microparticles adsorbed onto the cell surface with seemingly high affinity and induced apoptosis of cells, resulting in a significant reduction in cell proliferation and viability (Figs. 3, 4). These combined findings suggest that silica microparticles contained in plastic tubes for the purpose of blood coagulation may be hazardous where silica-contaminated PRF matrices are implanted [12]. Future research remains needed to better understand their use in clinical practice.

Clinical significance: In general, silica coatings actually have a high affinity for cell membrane walls, causing apoptosis and a reduction in cell proliferation.

### Study 4: Miron et al. (2021)—chemical additives to PRF tubes including silica and silicone negatively impacts final PRF clot size

**Fig. 5:**
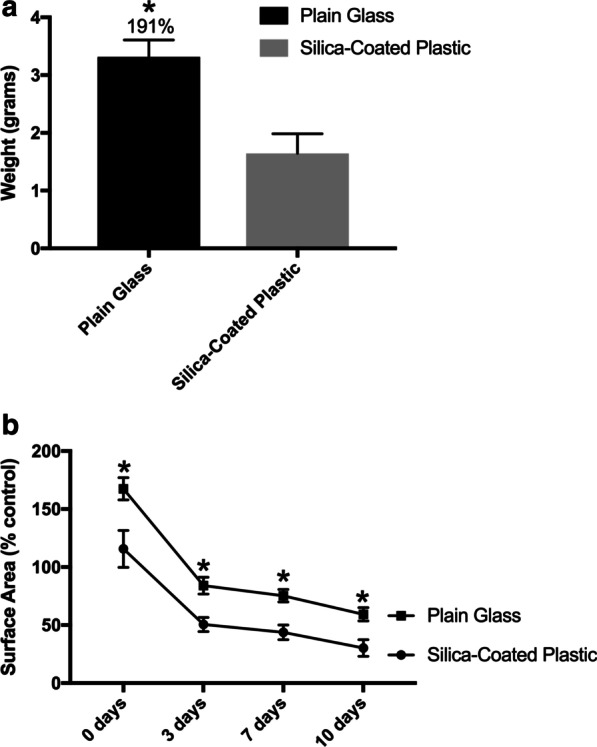
Comparative analysis of PRF tube clot weights and sizes from 6 individuals after centrifugation utilizing the same centrifugation speed and time and either plain glass tubes or silica-coated plastic tubes. **a** In general, the silica-coated plastic tubes on average decrease the final weight of PRF-based matrices nearly twofold. **b** Over a 10-day period, while both PRF clots are slowly and gradually degraded over time, significantly increased amounts of membrane remain at 0, 3 and 7 days. Both glass tubes and silica-coated plastic tubes were filled to 9 mL (Reprinted with permission from Miron et al. 2021 [13]

Following the group’s previous publication highlighted in study 1, a second study was performed investigating the effect of various sizes on the final size outcomes of PRF clots. Analysis over a 10-day period comparing the differences between PRF clots demonstrated that PRF clots produced in plastic silica-coated tubes were significantly smaller (roughly 2 times smaller) than those produced from glass tubes (Fig. 5a) [13]. Furthermore, trends continued favoring plain chemical-free glass tubes over the entire 10-day study period (Fig. 5b). It was further noted that much future research on tube chemistry is necessary to better understand clot formation, even formation in response to plain chemical-free glass tubes fabricated via different methods and starting materials.

**Fig. 6:**
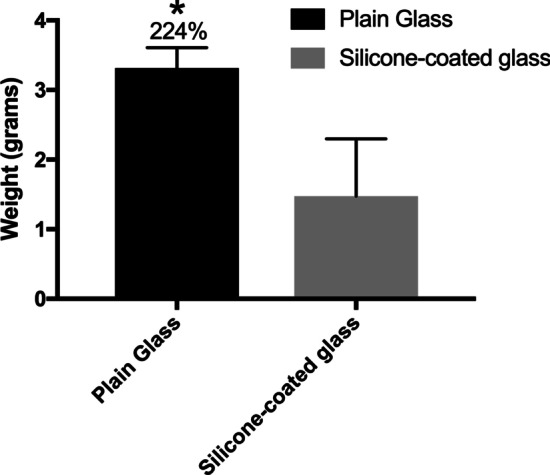
Effect of the addition of silicone to glass tubes. (A) Comparative weight analysis of PRF clots produced in plain glass tubes versus those produced in A-PRF glass tubes coated with silicone. Clot membranes increase more than 200% in plain glass tubes. Silicone may be more detrimental to standard clot size than silica (Reprinted with permission from Miron et al. 2021 [13]

More recently, the addition of silicone has specifically been introduced at high levels into A-PRF glass tubes. Comparative analysis revealed that PRF clots produced in A-PRF glass tubes containing silicone-coated walls had drastically reduced clot size formation (by over twofold) compared to the size of those produced with standard plain glass tubes (Fig. 6) [13]. Notably, some samples did not undergo any clotting after a standard 8 min centrifugation cycle. Silicone addition results in the production of a residue when either blood or water is introduced into PRF tubes.

Clinical significance: Both silica and silicone addition to the interior of PRF walls led to a nearly twofold reduction in PRF clot sizes. Furthermore, A-PRF silicone tubes specifically left a pronounced foam-like residue following interactions with solutions.

### Study 5: Takahashi et al. (2020)—distribution of platelets, transforming growth factor‐β1, platelet‐derived growth factor‐BB, vascular endothelial growth factor and matrix metalloprotease‐9 in advanced platelet‐rich fibrin and concentrated growth factor matrices

The aim of this study was to investigate the distribution of cells and growth factors in PRF following centrifugation in 2 different types of centrifugation devices [14]. To address this question, the authors compared A‐PRF and concentrated growth factor (CGF) matrices in terms of the distribution of platelets, transforming growth factor‐β1, platelet‐derived growth factor‐BB, vascular endothelial growth factor and matrix metalloprotease‐9 (MMP9). Blood samples were obtained in glass tubes and immediately centrifuged to prepare the A‐PRF or CGF matrix according to their specific protocols. Both matrices were compressed, embedded in paraffin and subjected to immunohistochemical examination [14].

**Fig. 7:**
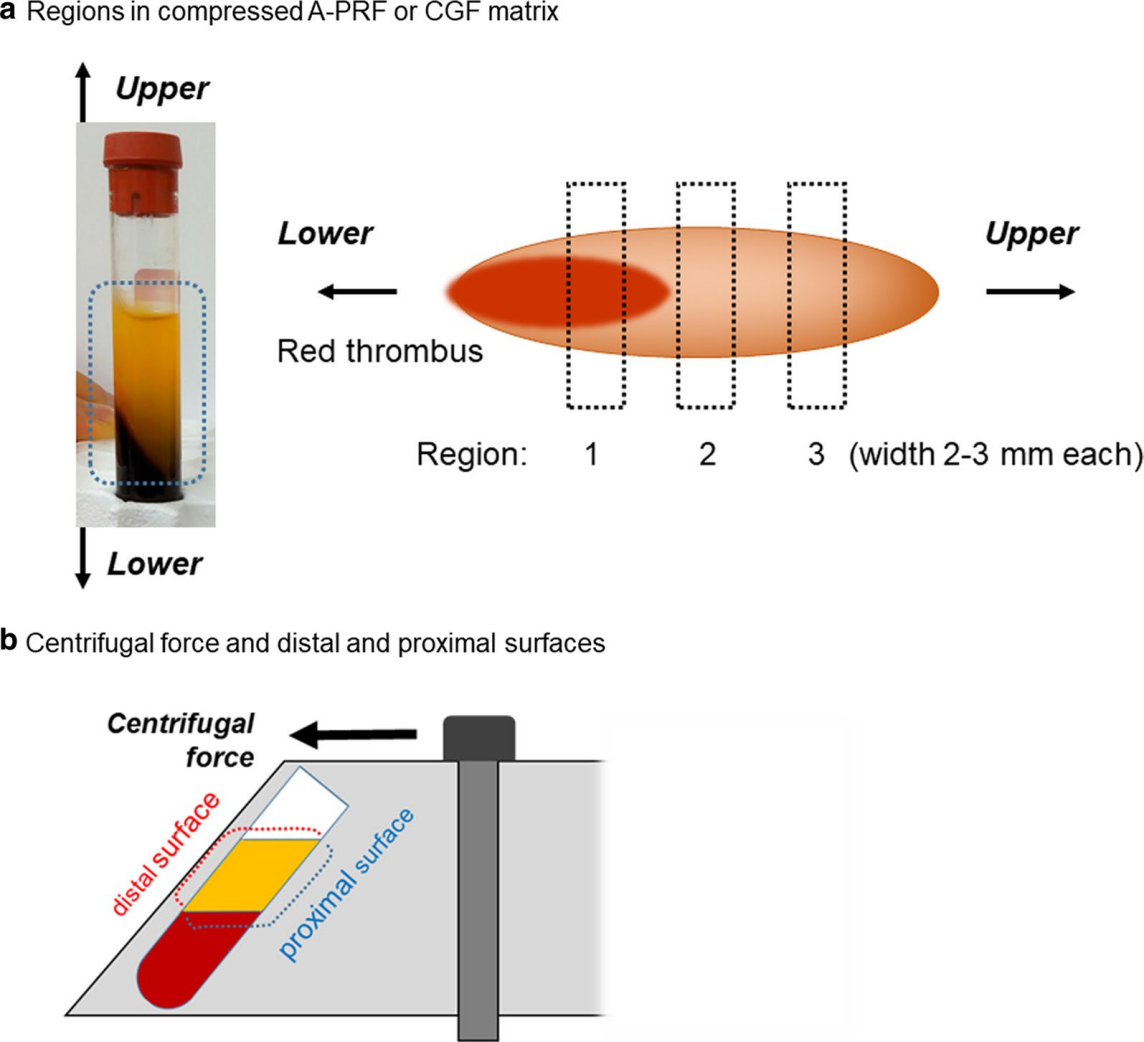
Experimental setup describing the orientation of PRF membranes during histological assessment. The proximal surface describes the inner tube wall (generally receiving the smallest g-force), whereas the distal surface is the outer tube wall, where cells generally accumulate during centrifugation at high g-force. **a** Regions in compressed A‐PRF or CGF matrix. This image is the proximal surface. **b** Centrifugal force and distal and proximal surfaces of A‐PRF or CGF matrix. A‐PRF, advanced platelet-rich fibrin; CGF, concentrated growth factors. Reprinted with permission from Takahishi et al. 2019 [14]

**Fig. 8:**
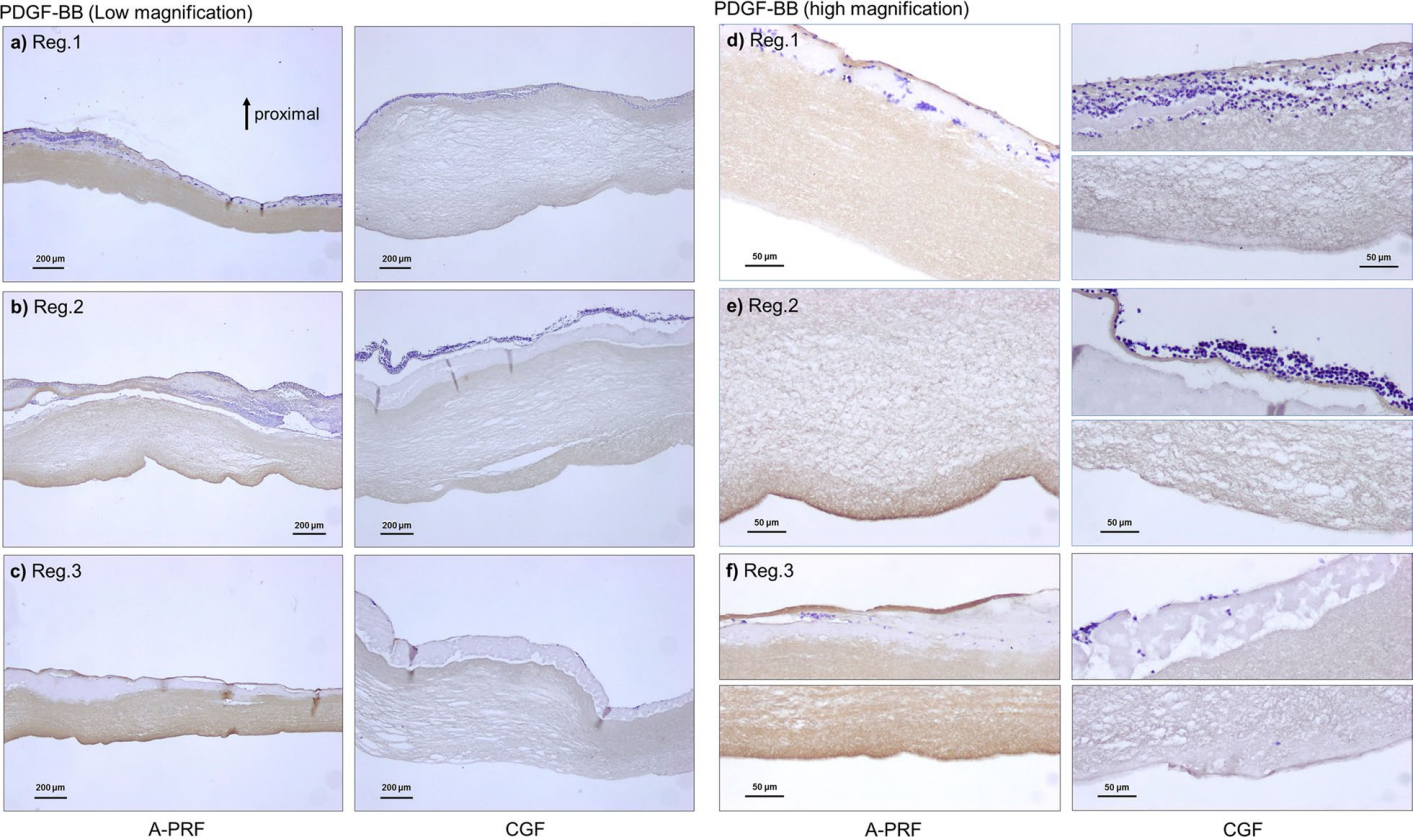
Distribution of PDGF‐BB in A‐PRF and CGF matrices. **a**, **d** Region 1, **b**, **e** region 2, and **c**, **f** region 3. **a**–**c** Low magnification, **d**–**f** high magnification. Note that the majority of cells and growth factor accumulate on the back distal surfaces of PRF tubes. A‐PRF, advanced platelet‐rich fibrin; CGF, concentrated growth factors; PDGF‐BB, platelet‐derived growth factor‐BB. Reprinted with permission from Takahishi et al. 2019 [14]

**Fig. 9:**
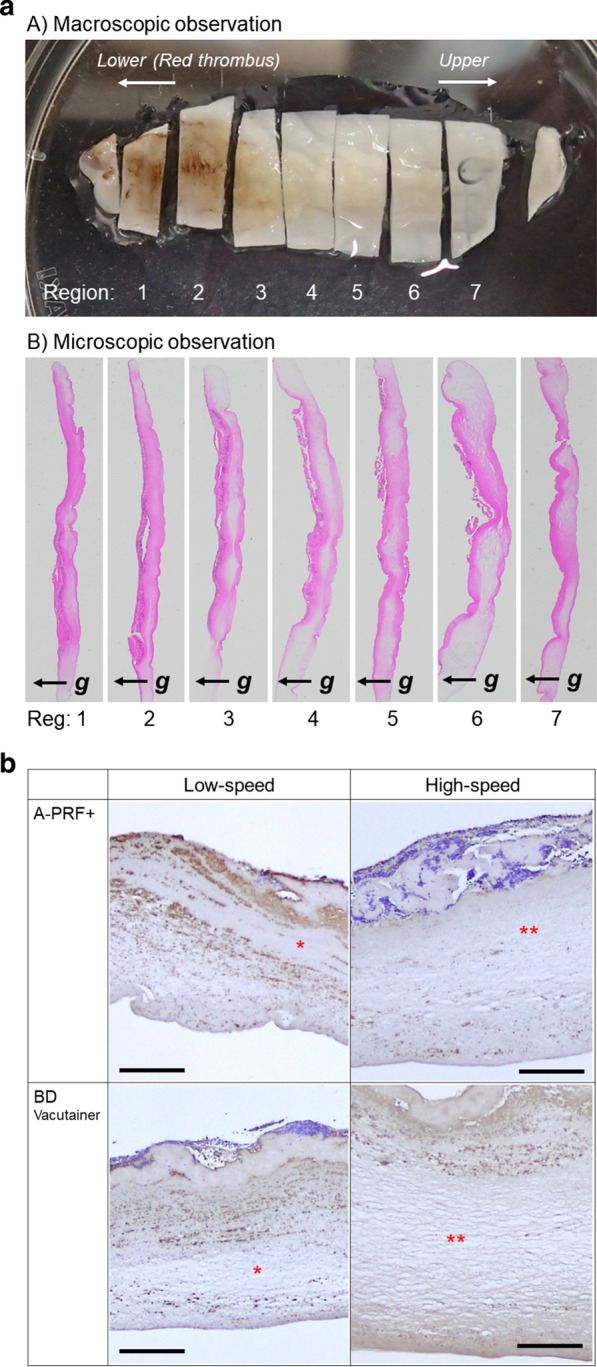
Macroscopic observation of a compressed and fixed A-PRF membrane. **a** This PRF membrane was divided into seven pieces, designated regions 1 to 7, where region 1 represents the region closest to the red blood cell fraction. **b** Microscopic observation of A-PRF cross-sections obtained from individual regions. Cross-sections were stained with hematoxylin and eosin (HE). **c** To confirm morphological similarity, the size of sections was modified to adjust their lengths at similar levels. Arrows represent the direction of gravity force. Summary of platelet distribution under various conditions. Asterisks represent wide-open spaces, and platelets were distributed sparsely. Double asterisks represent wider spaces than single asterisks. Bar scales are 200um. In general, it was found that cells were more evenly distributed at low centrifugation speeds, whereas high-speed centrifugation typically led to more cells accumulated at the back distal surfaces and closer to the buffy coat. Reprinted with permission from Tsujino et al. 2019 [15]

Following histological assessment, it was observed that leukocytes and plasma proteins were localized on the back walls of PRF tubes (distal surface), including the interface corresponding to the buffy coat (Figs. 7, 8) [14]. It was also found that some variability existed between the two devices investigated. In both cases, however, the majority of cells accumulated on the back distal surfaces of centrifugation tubes when PRF was produced in fixed-angle centrifuges. Even more intriguing, even the PRF tubes (plain glass tubes versus silica-coated plastic tubes) had a significant impact on the final distribution of cells found within PRF clots when the clots produced at different centrifugation speeds were investigated histologically (Fig. 9) [15].

Clinical significance: PRF membranes produced with fixed angle centrifugation accumulated cells along the back distal walls of centrifugation tubes. This was impacted by the tube type selected.

## Discussion

The goal of the present technical note was to provide an overview of recent research on PRF tubes and their impact on the final PRF clots and potential negative impact on tissue integration. Several studies have now been performed on the topic, highlighting the impact of silica/silicone coatings on either plastic or glass tubes and their potential toxic effect on human cells and/or inflammatory response when implanted in vivo. Generally, clinicians have for years been concerned with the choice of PRF centrifugation device, placing little importance on the impact of PRF tubes. With previously published studies over the past 1–2 years, we have convincingly shown that PRF tubes are more important than the actual centrifugation device utilized.

Unfortunately, much variability exists between tubes produced via various commercial entities with little respect placed on their quality, with virtually no research being performed on the topic to date. In 2018, a study by our group demonstrated convincingly that the PRF clots actually varied in size by as much as 250% between centrifugation companies, even when all centrifugation protocols were carried out on the same machine from blood from the same patient at the same protocol [11]. This led several research groups from around the world to further investigate the impact of centrifugation tubes on the final outcomes of PRF clots.

The group of Kawase in Japan obtained clear evidence that silica microparticles derived from commercially available blood collection tubes not only ‘leak’ within PRF tubes [10] but also exert toxic effects on human periosteal cells by adsorbing on the plasma membrane and inducing apoptosis. In addition, this cytotoxicity exceeded expectations, with silica microparticles contained in silica-coated tubes (e.g., Neotube and Vacuette) completely disrupting cell growth and viability.

It was reported in that study that amorphous silica is less toxic than crystalline silica. However, previous studies have demonstrated that amorphous silica is also hazardous to health. In the group’s previous study [10], it was demonstrated that 5–30% silica microparticles, depending on tube brands, were included in the resulting PRF clots. The collective data support the prediction that PRF preparations using silica-coated tubes could be toxic to the surrounding cells at implantation sites. During and after preparation of a PRF matrix, silica microparticles may also overactivate or disrupt platelets [16] and other blood cells in the PRF matrix and reduce their therapeutic potency and efficacy. Future research on this topic is needed, as there seems to be ways that are more effective to produce PRF without chemical additive incorporation into PRF tubes.

To our knowledge, however, there have been no complaints reporting severe complications from the application of silica-dependent PRF preparations. This is probably due to the body’s efficient clearance of silica microparticles by phagocytosis or extrusion, detoxification by scavengers [17], and/or cytoprotection by redox systems [18] and serum albumin [19]. Nevertheless, the additional incorporation of additives such as silica into PRF clots is not advantageous, especially granted the clot size is reduced twofold when compared to the size of clots produced with pure glass tubes.

Thus, even if no serious complications arise, delays in tissue regeneration or additional inflammatory responses may be possible. Lung silicosis, for example, is caused by chronic inhalation of silica dust for a prolonged period of time [20]. Since the clinical use of PRF matrices is much shorter than the duration of lung silicosis (several decades), it may be that short-term exposure to silica may not have long-term detrimental effects on the regenerated sites. However, the accumulation of DNA damage in cells, as observed in silicosis-derived lung cancer cells [20], may have little impact on cells involved in regenerative dentistry. Nevertheless, no biomedical merits are seen by using such tubes and PRF clots are nearly 2 times smaller when utilized. For these reasons, the authors recommend that clinicians not use these types of blood-collection tubes for the preparation of PRF.

As previously stated in a paper by Masuki et al. [12]: “To avoid misunderstanding, it must be noted that silica is different from silicone. In fact, a historical debate may have arisen because of this misunderstanding [21, 22]. The website provided by Stream Peak International concisely summarizes the terminology regarding silica, silicon and silicone. According to this website, silica, which is also known as silicon dioxide, is a compound that naturally forms in a reaction between oxygen and silicon. Silica is commonly used in the manufacturing of glass, ceramics, optical fiber, and cement. Silicon (Si) is the second most abundant element on Earth. However, it is rarely found in its original state as Si, as it readily reacts with oxygen to mainly form silicon dioxide. In contrast, silicone is a synthetic polymer created from the combination of silicon, oxygen, carbon, and/or hydrogen. Unlike natural materials that include silica and silicon, silicone is a man-made product that is manufactured in factories as a solid, a liquid, and a gel. Silicone is commonly used as sealant, electrical insulation, a component of cooking utensils, and a coating of test tubes. Therefore, even though silicone used for tube coatings may contain silica-like compounds, it cannot activate blood coagulation. Excess silicone coating actually delays coagulation. Furthermore, even if silicone has negative effects on the immune system and/or cells directly involved in tissue regeneration, these effects should be distinguished from those of silica. In any case, when platelet concentrates are prepared for use in regenerative therapy, we believe that real “plain” tubes that are approved by regulatory authorities of individual countries, regardless of their original materials, are better for clinical use.” [12]

Surprisingly, research conducted on the topic has actually demonstrated that the addition of silicone even in plain glass tubes led to a drastic ~ 200% reduction in PRF clot sizes (Fig. 6). Even plain glass tubes sold by BD for laboratory testing incorporate a layer of silicone within the inner tube walls (company website). These may be improved by removal of silicone. In summary, it is highly recommended that treating clinicians be aware of the great impact that centrifugation tubes play on the final outcomes of PRF-based matrices and that the addition role that chemical additives may play on the final PRF clot outcomes.

## Conclusions

The present overview article highlights the importance of PRF tubes with respect to the final outcomes of PRF clots. These authors note that it is important to pay attention to the quality of the devices (e.g., registration or approval as a medical device by regulatory authorities) and somewhat ‘hidden’ chemical additives in PRF tubes. For example, silica‐coated plastic tubes, which are increasingly used as alternatives to glass tubes, produce a distinguishable type of fibrin matrix in terms of platelet distribution and contamination by silica particles that negatively impact cell survival and proliferation. Similarly, the addition of silicone to glass tubes has further been reported to cause increased inflammatory responses in humans with delays in clot formation following standard centrifugation protocols. The goal of the present article was to alert clinicians of potential risks for using tubes containing chemical additives for the production of PRF since they may interfere or negatively impact tissue regeneration at implantation sites. Although vigorous debates among certain colleagues sometimes produces substantial bias, the authors caution that further research, particularly with plain non-chemical tubes, should be the focus of intensive research efforts from a neutral standpoint over the coming years to substantially progress PRF therapy.

## Data Availability

The datasets used and/or analyzed during the current study available from the corresponding author on reasonable request.

## Abbreviations

A-PRF: Advanced Platelet Rich Fibrin
BD: Becton Dickinson
CGF: Concentrated Growth Factor
H&E: Hematoxylin and Eosin
MMP9: Matrix Metalloprotease‐9
PRF: Platelet Rich Fibrin
PDGF: Platelet Derived Growth Factor
PRF: Platelet Rich Fibrin
PRP: Platelet Rich Plasma
SEM: Scanning Electron Microscopy
Si: Silicone
TGF-β: Transforming Growth Factor-beta
VEGF: Vascular Endothelial Growth Factor
WBC: White Blood Cell
